# Supplementary material for: Off-Hour Admission Is Associated with Poor Outcome in Patients with Intracerebral Hemorrhage
Source: J Clin Med. 2022 Dec 21;12(1):66. doi: 10.3390/jcm12010066 (PMC9821144; doi:10.3390/jcm12010066)
Supplement: Supplementary file 1 [file jcm-12-00066-s001.zip › jcm-2053501-supplementary.pdf]

Journal of
Clinical Medicine

MDPI

Supplemental Material.

Table S1. Collinearity diagnostic as follow.

Variable	Tolerance	VIF
Age, year	0.988	1.012
Systolic blood pressure, mmHg	0.921	1.085
Admission GCS score	0.752	1.329
Baseline ICH volume, mL	0.968	1.033
IVH at baseline CT	0.790	1.266
Time from onset to CT, hour	0.903	1.107
Off-hour admission	0.964	1.037

GCS: Glasgow Coma Scale, ICH: Intracerebral hemorrhage, CT: computed tomography, IVH: intraventricular hemorrhage, VIF: variance inflation factor.
